# Multimode Miniature Polarization-Sensitive Metamaterial Absorber with Ultra-Wide Bandwidth in the K Band

**DOI:** 10.3390/mi15121446

**Published:** 2024-11-29

**Authors:** Zhonghang Ji, Yida Song, Mandi Gao, Qiong Zhang, Yunqing Liu

**Affiliations:** 1 College of Electronic Information Engineering, Changchun University of Science and Technology, Changchun 130022, China; yida@mails.cust.edu.cn (Y.S.); mandi@mails.cust.edu.cn (M.G.); zhangqiong15@mails.jlu.edu.cn (Q.Z.); mzliuyunqing@163.com (Y.L.); 2 Jilin Provincial Science and Technology Innovation Center of Intelligent Perception and Information Processing, Changchun 130022, China

**Keywords:** metamaterial absorber, ultra broadband, multifunctional, polarization sensitive, K band

## Abstract

Metamaterial absorbers have gained widespread applications in fields such as sensing, imaging, and electromagnetic cloaking due to their unique absorption characteristics. This paper presents the design and fabrication of a novel K-band polarization-sensitive metamaterial absorber, which operates in the frequency range of 20.76 to 24.20 GHz for both TE and TM modes, achieving an absorption rate exceeding 90% and a bandwidth of up to 3.44 GHz. The structure of the metamaterial absorber consists of a rectangular aperture metallic patch, two metallic rings, and two metallic strips, with a metallic patch structure on the back. Both metallic patches are printed on a 1.575 mm-thick FR-4 substrate. In the TE mode, the performance shows diagonal symmetry, with a minimum absorption bandwidth of 1.4 GHz at 45° and a maximum of 3.44 GHz at 0°. The absorption rate exceeds 90% across various polarization angles. In terms of conventional modes, both the TE and TM modes can achieve ultra-wideband absorption. For specific scenarios requiring single-frequency or multi-frequency absorption, the desired functionality can be realized by varying the incident angle. These exceptional characteristics confer strong applicability for high-bandwidth electromagnetic wave absorption and specific frequency point absorption, indicating significant potential and practical value in the field of wireless communication.

## 1. Introduction

Metamaterials possess unique physical properties that distinguish them from natural materials. Metamaterial absorbers offer advantages such as a lightweight design, thin profiles, and high absorption rates, making them widely applicable in engineering fields, including in lenses [1,2,3,4], cloaking devices [5,6,7,8], holographic imaging [9,10,11,12], and electromagnetic wave shielding [13,14,15,16]. Since the development of the first absorber by Landy et al. [17], numerous metamaterial electromagnetic wave absorbers have been created to meet specific absorption characteristics across targeted electromagnetic wave ranges. Over time, researchers have introduced dual-layer [18,19] and multilayer metamaterial absorbers [20,21,22]. For instance, Yongqiang Pang [23] and his team developed a metamaterial absorber based on high-impedance surfaces, while Zhiqiang Du [24] and his team created a low-frequency lumped resistance structure, with their absorption bands covering the C-band and the L to S bands, respectively. In the microwave frequency range, numerous studies have reported single-band and multi-band metamaterial absorbers. Notably, designs covering the C and X bands [25,26], as well as three-band absorbers for the X, Ku, and K bands [27,28], have emerged. Saif Hannan et al. [29] proposed a segmented split-ring resonator symmetric structure, achieving absorption across the X, Ku, and K bands with a polarization-insensitive characteristic and an absorption bandwidth of approximately 1 GHz in the K band. Adnan Yousaf et al. [30] introduced an ultra-thin metamaterial absorber with a thickness of only 0.65 mm, also covering the X, Ku, and K bands, achieving over a 90% absorption bandwidth in the K band around 1 GHz, even under a 70° oblique incident electromagnetic wave. Mohammad Lutful Hakim et al. [31] presented a three-band metamaterial absorber that incorporates a modified split-ring resonator structure with a cross-shaped element at its center. This design exhibits single-negative characteristics (SNG) at 21.6 GHz and double-negative characteristics (DNG) at 24.04 GHz, achieving over 90% absorption in two K-band frequency ranges: 21.4 GHz to 21.8 GHz (400 MHz) and 23.84 GHz to 24.24 GHz (760 MHz), while remaining polarization insensitive. This research offers a wider bandwidth absorption characteristic in the K band compared to previous designs, making it a promising candidate for applications in Bluetooth, filtering, and sensing. However, the absorption bands are segmented into two narrow ranges rather than a continuous bandwidth, limiting the ability to achieve high-bandwidth absorption across a continuous frequency range. Therefore, the design of a metamaterial absorber with miniaturization, high bandwidth, and improved absorption performance remains a pressing problem.

This paper proposes a K-band metamaterial absorber that achieves ultra-wideband electromagnetic wave absorption in both the TE and TM modes, demonstrating over 90% absorption in a continuous frequency range from 20.76 GHz to 24.20 GHz (3.44 GHz). In this work, a method for calculating the relative bandwidth for absorption (RBA) is introduced, defined as the ratio of the electromagnetic wave absorption bandwidth to the entire frequency range. The RBO of the proposed absorber is up to 43%. Considering the compactness and complexity of its structures, the proposed metamaterial absorber holds great potential for K band applications. In TE mode, the absorption rate remains above 90% for various polarization angles, although the corresponding absorption bandwidth varies. At a polarization angle of 45°, the absorption bandwidth is at its narrowest at 1.4 GHz, whereas at 0°, it reaches its widest bandwidth at 3.44 GHz. In TM mode, the absorption rates at 0° and 90° are identical to those in TE mode, while other angles exhibit rates below 80%. Additionally, in TM mode, oblique incident electromagnetic waves at angles between 15° and 75° show absorption characteristics at specific frequency points. Detailed data on polarization sensitivity will be discussed further in the paper. To validate the performance of the proposed structure, simulations were conducted using CST STUDIO SUITE, and its equivalent circuit was analyzed using ADS 2021 software to confirm its feasibility. Effective parameters such as permeability, permittivity, and refractive index were also analyzed. Systematic simulations of the electric field, magnetic field, and current distribution were performed to corroborate the high-bandwidth absorption characteristics. Finally, physical testing of the metamaterial absorber was carried out, and the results were compared with simulation data, showing complete agreement. Both experimental and simulation results indicate that the proposed K-band absorber holds significant potential for ultra-wideband electromagnetic wave absorption and selective frequency absorption, showcasing considerable promise and cutting-edge applications in the field of wireless communication.

## 2. Materials and Methods

### 2.1. Unit Cell Design

**Figure 1:**
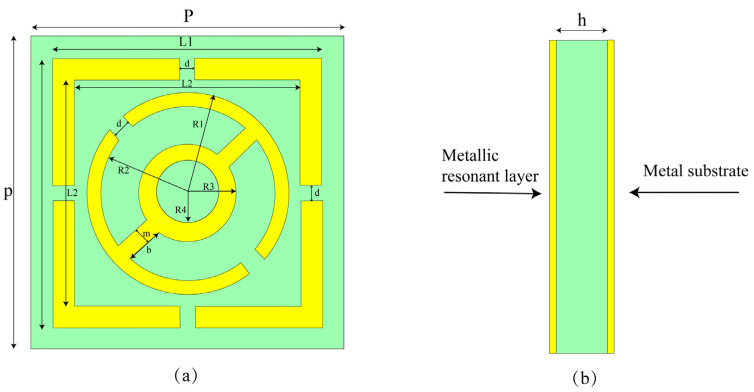
(**a**) Front view of the metamaterial. (**b**) Side view of the metamaterial.

In this section, we detail the design steps of the unit cell, the absorption mechanisms, and its equivalent circuit. The substrate material used was FR-4, with a thickness of 1.575 mm and a dielectric constant of $\varepsilon_{r}$ = 4.3. The structure features a double-sided metallic patch configuration, with both sides printed on a 9 mm × 9 mm FR-4 substrate, with a copper thickness of 0.035 mm. The front and side views are shown in Figure 1a,b, respectively, and specific dimensions are provided in Table 1. The absorption performance of the absorber can be calculated using the characteristic equation, as follows [32]:

(1) $$A=1-R(\omega )-T(\omega )=1-|S_{11}{|}^{2}-|S_{21}{|}^{2}$$

Here, $R(\omega )={\left|S_{11}\right|}^{2}$ and $T(\omega )={\left|S_{21}\right|}^{2}$ represent the reflection power and transmission power, respectively. Due to the metal backplane of the structure acting as a barrier to the transmission of electromagnetic waves, it serves as a physical shield against electromagnetic wave transmission; therefore, the transmission coefficient $S_{21}$ is approximately 0. The characteristic equation for absorption performance can be simplified as follows:


(2) $$A=1-R(\omega )=1-|S_{11}{|}^{2}$$

**Figure 2:**
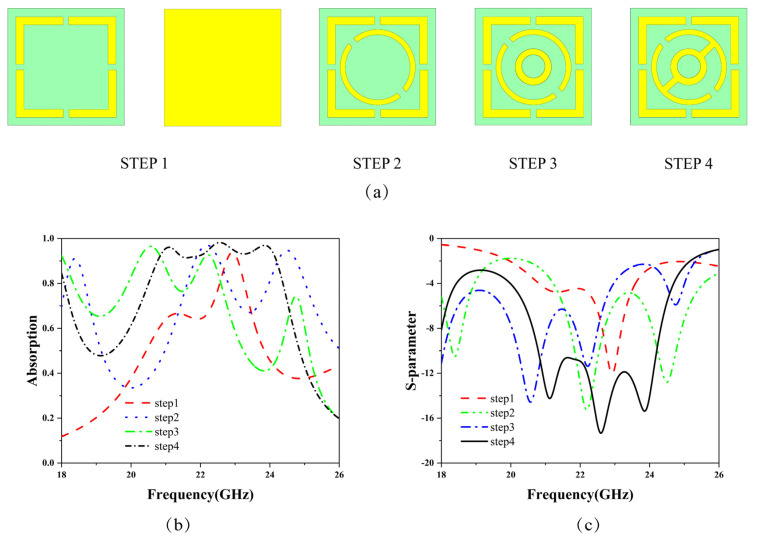
(**a**) Steps for absorber design. (**b**) Absorption rate curves for each step. (**c**) $S_{11}$ curves for each step.

The specific design steps for the structure are illustrated in Figure 2a. For the metal–dielectric–metal metamaterial absorber, the design concept presented in this paper aims to achieve ultra-wideband absorption based on the frequency point absorption studied in previous research. The rectangular metallic resonant rings in Step 1 correspond to low-frequency absorption points, which can be equivalently modeled as an RLC circuit in the equivalent circuit. Through Step 2 and Step 3, multi-frequency point absorption is realized. Then, through electromagnetic coupling characteristics, the coupling of the resonant points is enhanced. In Step 4, rectangular metallic patch structures are introduced to further enhance the coupling between the three resonant points, thus achieving ultra-wideband absorption. The absorption rates and S11 curves for each step are shown in Figure 2b,c. According to the absorption rate curve from the simulation in Step 4 shown in Figure 2b, the structure demonstrates an absorption rate exceeding 90% in the frequency range of 20.24–23.20 GHz. Additionally, there are three absorption peaks at 21.12 GHz, 22.60 GHz, and 23.86 GHz, with absorption rates of 96.24%, 98.16%, and 97.10%, respectively.

### 2.2. Equivalent Circuit

According to transmission line theory, the structure of a metamaterial absorber can be equivalently modeled using inductance, capacitance, and resistance. In the circuit model, the metallic patches can be viewed as inductive elements, while the gaps between the patches can be equivalent to capacitance. The losses generated during transmission and reflection processes can be regarded as resistance. For a metamaterial absorber with a metallic backplane, its equivalent circuit can be considered as a single-port network. By fitting the circuit using an R–L–C series configuration, a resonant point can be established, while a parallel configuration of three sets of R–L–C series can effectively construct the equivalent circuit of the metamaterial absorber. Using ADS software, the values of R, L, and C can be adjusted to simulate the S-parameters. Additionally, the resonant frequency of the LC circuit, as a type of oscillating circuit, can be determined using the following formula:

(3) $$f=\frac{1}{2\pi \sqrt{LC}}$$

Here, *L* represents the inductance, and *C* denotes the capacitance. The essence of fitting the equivalent circuit of the metamaterial absorber is based on transmission line theory. According to the principles of transmission lines, the equivalent inductance can be calculated [33]:

(4) $$L=0.01\times \mu_{0}\left\{\frac{2{\left(d+g+h\right)}^{2}}{{\left(2w+g+h\right)}^{2}}+\frac{\sqrt{{\left(2w+g+h\right)}^{2}+p}}{\left(d+g+h\right)}\right\}t$$

According to the principles of transmission line theory, the equivalent capacitance can be calculated using the following formula:

(5) $$C=\varepsilon_{0}\left[\frac{2w+g+h}{2\pi {\left(d+h\right)}^{2}}\ln\frac{2(d+g+h)}{\left(a-l\right)}\right]t$$

**Figure 3:**
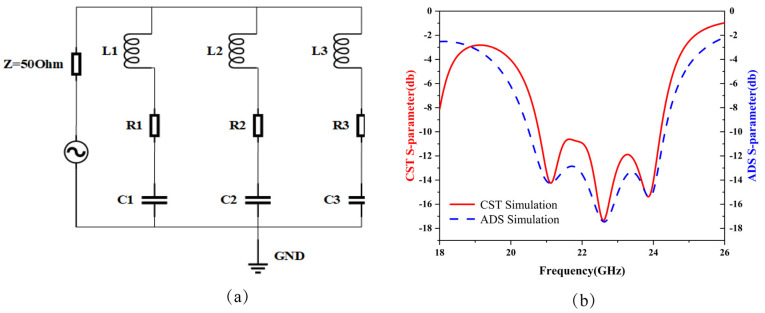
(**a**) Equivalent circuit diagram. (**b**) Comparison of ADS and CST simulation results.

Here, *w* represents the width of the metallic patch, *h* denotes the thickness of the substrate, *t* indicates the thickness of the metallic patch, *l* is the length, *d* is the distance between the opening of the resonant ring, *g* is the gap distance between resonant units, and *a* is the boundary dimension of the structure. The comparison between the circuit diagram and the simulated S-parameters from CST 2023 software is shown in Figure 3a,b. The specific equivalent parameters are as follows: L1 = 4.6 nH, L2 = 5.3 nH, L3 = 5.52 nH, R1 = 32.2 Ω, R2 = 43.9 Ω, R3 = 40.6 Ω, C1 = 0.0129 pF, C2 = 0.00905 pF, and C3 = 0.0077 pF.

### 2.3. Effective Parameter Analysis

To further analyze the absorption mechanism, the electromagnetic characteristics of the structure were examined using the reflection coefficient $S_{11}$ and transmission coefficient $S_{21}$. The effective parameters, including permittivity, permeability, refractive index, and normalized impedance, were calculated using the robust retrieval method. The equations for the reflection coefficient $S_{11}$ and transmission coefficient $S_{21}$ are shown below [34].

(6) $$S_{11}=\frac{R_{01}(1-e^{i2nk_{0}d})}{1-R_{01}^{2}e^{i2nk_{0}d}}$$

(7) $$S_{21}=\frac{\left(1-R_{01}^{2}\right)e^{ink_{0}d}}{1-R_{01}^{2}e^{i2nk_{0}d}}$$

where

(8) $$R_{01}=z-1/z+1$$

(9) $$e^{i2nk_{0}d}=\frac{S_{21}}{1-S_{11}\frac{z-1}{z+1}}$$

The impedance is as follows:

(10) $$z=\sqrt{\frac{{\left(1+S_{11}\right)}^{2}-S_{21}^{2}}{{\left(1-S_{11}\right)}^{2}-S_{21}^{2}}}$$

The refractive index is as follows:

(11) $$n=\frac{1}{k_{0}d}cos^{-1}\left[\frac{1}{2S_{21}}(1-S_{11}^{2}+S_{21}^{2})\right]$$

Here, the real part is denoted by ()′ and the imaginary part by ()″. *m* represents the integer part of the real part of the refractive index, and $k_{0}$ is the spatial frequency of the incident electromagnetic wave in free space, which is the wave number; the wave number is the reciprocal of the wavelength *λ*. *d* is the thickness of the substrate, so the refractive index can also be expressed as the following:

(12) $$n=\frac{1}{k_{0}d}[\ln{\left(e^{i2nk_{0}d}\right)}^{\prime \prime }+2m\pi ]-i[\ln{\left(e^{i2k_{0}d}\right)}^{\prime }]$$

The permeability is as follows:

(13) μ=nz

The permittivity is as follows:

(14) ε=n/z

**Figure 4:**
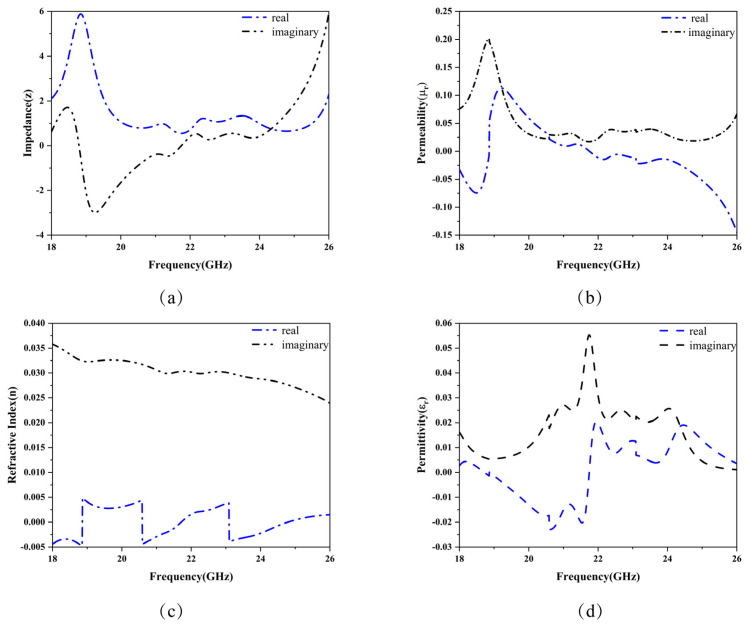
The effective parameters of the metamaterials in the frequency range of 18 GHz to 26 GHz. The blue dashed line represents the real part, and the black dashed line represents the imaginary part. (**a**) Normalized impedance. (**b**) Permeability. (**c**) Refractive index. (**d**) Permittivity.

**Figure 5:**
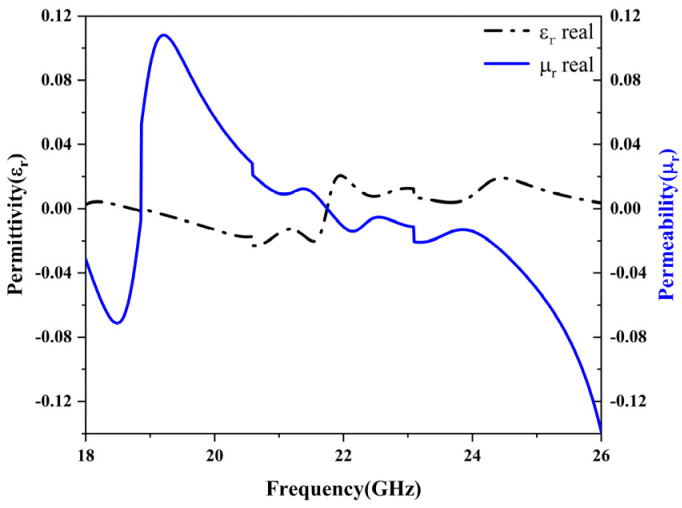
An illustration of the real parts of the permittivity and permeability in the frequency range of 18 GHz to 26 GHz. The black dot–dash line represents the real part of the permittivity, and the blue line represents the real part of the permeability.

The effective parameters are shown in Figure 4, and a comparison of the magnetic permeability and the real part of the permittivity is illustrated in Figure 5. The real part of the permittivity $\varepsilon_{r}$ represents the propagation characteristics of the electric field within the material, while the imaginary part indicates the material’s loss characteristics. Higher loss corresponds to greater absorption of electromagnetic waves by the material. Similarly, the real part of the permeability $\mu_{r}$ reflects the material’s response to the magnetic field, whereas the imaginary part represents magnetic loss, indicating energy dissipation under the influence of the magnetic field.

As shown in Figure 4b,d, in the frequency range of 20.76–21.79 GHz, the permittivity $\varepsilon_{r}$ is positive, and the permeability $\mu_{r}$ is negative. The negative permeability suppresses the propagation of the magnetic field, leading to a wave attenuation effect. In the frequency range of 21.79–24.20 GHz, the permittivity $\varepsilon_{r}$ becomes negative, while the permeability $\mu_{r}$ is positive. In this case, the material exhibits a negative response to the electric field but a positive response to the magnetic field, preventing the effective propagation of electromagnetic waves and resulting in rapid attenuation.

As illustrated in Figure 5, the alternating signs of the real parts of $\varepsilon_{r}$ and $\mu_{r}$ in the frequency range of 20.76–24.20 GHz contribute to the broadband absorption characteristics of the structure, achieving an absorption bandwidth of 3.44 GHz. Figure 4a depicts the real and imaginary parts of the normalized impedance, where the real part is close to 1, and the imaginary part is near 0, achieving impedance matching. According to formula [10], impedance matching minimizes the reflection coefficient $S_{11}$.

Additionally, as shown in Figure 4b,d, the imaginary parts of $\varepsilon_{r}$ and $\mu_{r}$ are positive throughout the studied frequency range, further confirming the presence of material loss. This observation is validated by the positive imaginary part of the refractive index, indicating the attenuation of electromagnetic waves within the material.

## 3. Surface Current, Electric Field, and Magnetic Field Analysis

Maxwell’s equations provide a detailed explanation of the relationships between the electric field (E-field), magnetic field (H-field), and surface current distribution in the metamaterial absorber, as expressed by the following equations:

(15) $$\nabla \times H=J+\epsilon \frac{\partial D}{\partial t}$$

(16) $$\nabla \times E=-\frac{\partial B}{\partial t}$$

The relationship between the electric field and the current density is given as follows:

(17) J=σE

In this context, *E* and *H* represent the electric field intensity and magnetic field intensity, respectively, while *D* and *B* denote the electric displacement vector and magnetic induction, respectively. *J* represents the current density.

**Figure 6:**
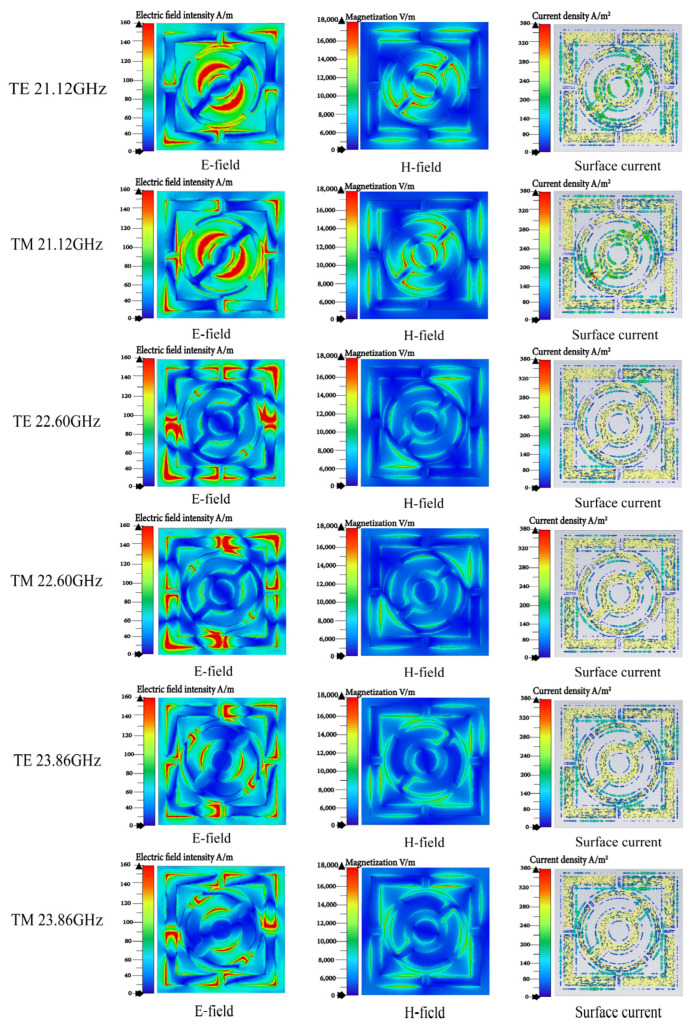
From left to right are the electric field, magnetic field, and surface current for the TM mode and TE mode, respectively.

To further investigate the absorption mechanism, simulations of the magnetic field, electric field, and current distribution at the absorption peaks of 21.12 GHz, 22.60 GHz, and 23.86 GHz, with absorption rates of 96.2%, 98.6%, and 97.7%, respectively, were conducted under TE and TM modes, as shown in Figure 6.

At 21.12 GHz, the electric field and magnetic field intensities exhibit high values between the smallest resonant ring and the gap with the outer resonant ring, indicating strong electromagnetic response at this frequency. Additionally, the surface current density is high, showing a clear current distribution that aligns with the electric field intensity distribution. This current distribution further confirms the structure’s effective electromagnetic absorption capability at this frequency. At 22.60 GHz, strong electromagnetic coupling is observed at the surface metal openings, particularly at the openings of the rectangular resonant rings, which is also reflected in the current distribution. For 23.86 GHz, the electric field intensity is similar to that at 22.60 GHz, but with a stronger magnetic intensity, confirming significant electromagnetic coupling and enhanced absorption capabilities. From Figure 6, it can be observed that the electric field intensity is consistent with the current distribution, while the magnetic field is perpendicular to the electric field. Thus, the magnetic field intensity and electric field intensity display different variation trends, which also corroborates the strong electromagnetic coupling characteristics at the three absorption peaks. In both the TE and TM modes, the electric field intensity, magnetic field intensity, and current distribution show negligible differences, validating the insensitivity of the absorber structure to both modes when electromagnetic waves are incident perpendicularly.

## 4. Analysis of Polarization and Incident Angle Effects on Absorber Behavior

**Figure 7:**
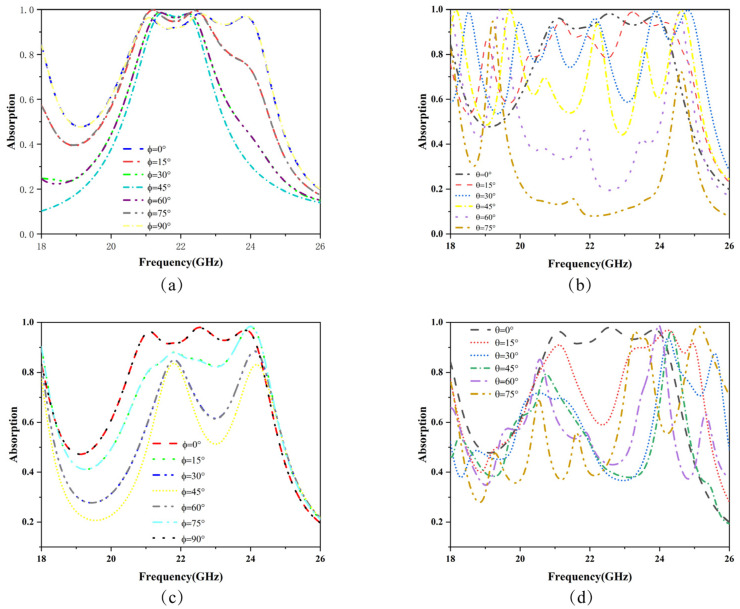
Different polarization angles and oblique incidence absorption curves for TE and TM modes: (**a**) TE mode with varying polarization angles. (**b**) TE mode with oblique incidence of electromagnetic waves. (**c**) TM mode with varying polarization angles. (**d**) TM mode with oblique incidence of electromagnetic waves.

This section investigates the absorption rates of the metamaterial absorber under different polarization angles and incident angles. In TE mode, when electromagnetic waves are incident in a fixed direction, the magnetic field component lies in the xy-plane, while the electric field component is perpendicular to the propagation direction, forming a perpendicular relationship between the electric and magnetic fields. Upon rotating the structure by 90°, the absorption range exhibits 45° symmetry, enhancing the flexibility of the metamaterial for practical applications. At different polarization angles, both 15° and 75° show an absorption range of 20.77 GHz to 22.90 GHz (2.13 GHz), while at 45°, it ranges from 21.08 GHz to 22.48 GHz (1.4 GHz), and at 30° and 60°, it is the same, at 21 GHz to 22.58 GHz (1.58 GHz). The absorption ranges for 0° and 90° are identical, with a maximum absorption bandwidth of 3.44 GHz, as shown in Figure 7a. Due to the 45° symmetry of the structure, the absorption bandwidth of the metamaterial varies with the polarization angle. With small changes in polarization angle, the high-frequency absorption peaks exhibit redshift, while low-frequency peaks display blueshift, most notably at 45°. As the angle continues to increase, the absorption bandwidth gradually shows a symmetrical pattern.

In TE mode, CST software was used for the simulation analysis of electromagnetic wave incident angles from 0° to 75°, with results shown in Figure 7b. According to multiple scattering reflection theory, the surface metal patch acts as a reflective structure, while the substrate acts as a transmissive structure. When electromagnetic waves pass through the substrate to the underlying metallic base, reflection occurs, promoting electromagnetic wave absorption. Due to the asymmetry of the absorber structure, transmission and reflection are not fully symmetrical. Based on transmission line theory, the directional impedance varies with the angle of the incident electromagnetic wave, leading to significant differences in absorption efficiency. When electromagnetic waves strike the medium, they create a plane, and different incident angles, according to the Doppler effect, cause changes in the equivalent impedance. These changes affect electromagnetic characteristics, thus influencing absorption and reflection. This unique property opens up more possibilities for the practical applications of metamaterials. Due to these differences, the electromagnetic absorption characteristics shift from continuous frequency absorption to multi-frequency absorption peaks within the K band. Specifically, at a 15° incident angle, three absorption peaks are observed at 21.17 GHz, 23.16 GHz, and 24.20 GHz. At 30°, six absorption peaks are observed in the K band at 18.50 GHz, 19.97 GHz, 20.91 GHz, 22.11 GHz, 23.88 GHz, and 24.78 GHz. At 45°, there are four absorption peaks at 18.14 GHz, 19.69 GHz, 22.19 GHz, and 24.60 GHz. At 60°, three absorption peaks are found at 18.01 GHz, 19.41 GHz, and 24.54 GHz, while at 75°, one absorption peak is observed at 19.21 GHz. These differences in absorption peaks at various angles demonstrate the practical value of this structure for filtering and high-performance antennas, adapting to the demands of different application scenarios.

For TM mode, when electromagnetic waves are incident in a fixed direction, the electric field component lies in the xy-plane, and the magnetic field component is perpendicular to the propagation direction, creating a perpendicular relationship between the magnetic and electric fields. The rotation angle of the material significantly impacts the absorption bandwidth across different polarization modes. Within the range of 0° to 90°, the absorption bandwidth at 0° and 90° overlaps and is consistent with the TE mode simulation results, indicating that the structure is insensitive to both the TE and TM modes at these angles. In the lower-frequency region, a blueshift in the absorption peaks is observed, as shown in Figure 7c, with only a low-bandwidth absorption peak at 15°, while the absorption efficiency at other angles is generally below 80%. In TM mode, the transmission and reflection of electromagnetic waves can be equivalently represented as impedance, according to transmission line theory. As the incident angle changes, the impedance of the asymmetric structure also changes, with significant differences between the TE and TM modes in different directional components, leading to variations in the inclined incidence absorption curves. As shown in Figure 7d, specific frequency absorption phenomena occur at certain angles in TM mode. For instance, at 15°, two absorption peaks are observed at 24.20 GHz and 24.89 GHz; at 30°, one absorption peak is located at 24.22 GHz; at 45°, the peak is at 24.32 GHz; at 60°, it is at 23.95 GHz; and at 75°, three absorption peaks are observed at 23.28 GHz, 23.56 GHz, and 25.06 GHz. The varying absorption frequencies corresponding to different incident angles in TM mode indicate that the absorber demonstrates excellent filtering performance, offering significant application value for single or multi-frequency absorption scenarios.

## 5. Measurement Results and Discussion

**Figure 8:**
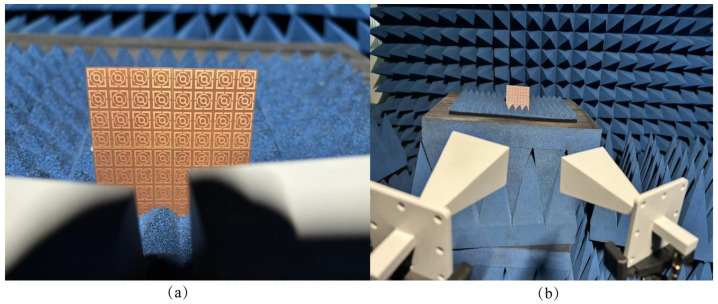
(**a**) Physical structure (**b**) Testing environment.

**Figure 9:**
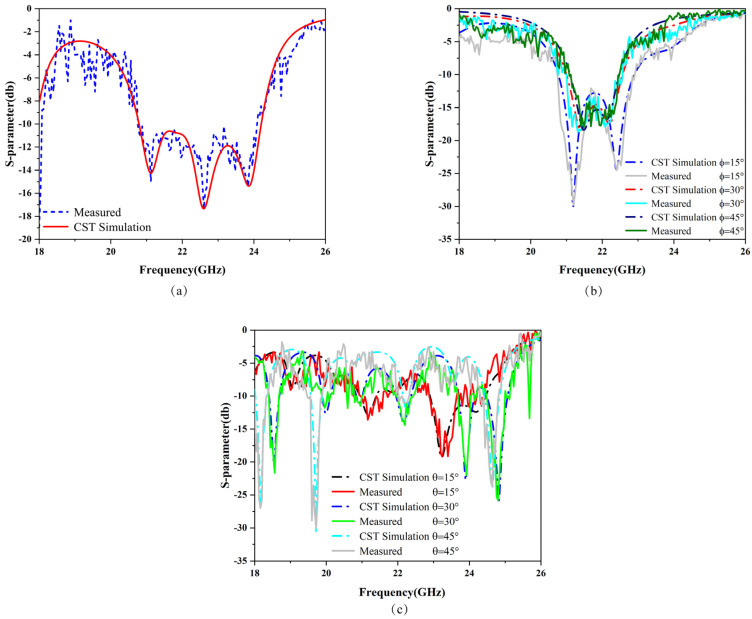
Experimental and simulation results of 18–26 GHz frequency range. (**a**) Normal incidence of electromagnetic waves; blue dashed line represents experimental results, and red solid line represents simulation results. (**b**) Experimental and simulation results for different polarization angles; dash–dot line represents simulation results, and solid line represents experimental results. (**c**) Experimental and simulation results for oblique incidence; dash–dot line represents simulation results, and solid line represents experimental results.

To verify the absorption characteristics of the proposed metamaterial absorber in the K band, physical measurements of the absorber structure were conducted and compared with simulation results. As shown in Figure 8, the metamaterial absorber structure utilizes printed circuit board (PCB) technology, featuring an 8 × 8-unit cell configuration with overall dimensions of 72 mm × 72 mm × 1.645 mm. The physical structure is depicted in Figure 8a, while the testing environment and measurement setup are illustrated in Figure 8b. The experiment utilized the Agilent Technologies N5230A Vector Network Analyzer (VNA) as the signal source to measure the S-parameters of the metamaterial absorber. A horn antenna, with a frequency range of 18 GHz to 26 GHz, was employed as both the transmitting and receiving element for the signal. First, a single broadband horn antenna was placed in an anechoic chamber to test the fully metallic backplane of the metamaterial absorber, serving as an ideal reflective structure. Subsequently, the front structure was tested using a spontaneous transmission and reception configuration, with a broadband horn antenna connected to a vector network analyzer to observe its S-parameters. Following this, two broadband horn antennas were employed for testing, allowing a comparison with the results obtained from the single antenna, verifying their S-parameters and absorption characteristics. One horn antenna acted as the transmitter and the other as the receiver, both connected to a vector network analyzer. Prior to testing the structure, the same procedure was repeated: first, testing the full metal back as the ideal reflective structure, followed by testing the front structure to achieve optimal results. After comparing the two test outcomes, it was observed that, during the transmission and reception of electromagnetic waves, the antenna beam experienced some path loss. The measured S-parameters of the absorber under normal incidence matched well with the S-parameters obtained from CST simulations, and the results are shown in Figure 9a. The evaluation of experimental and simulation results for different polarization angles and oblique incidence angles is shown in Figure 9b,c. The proposed metamaterial absorber was compared with previously reported K-band absorbers in terms of absorption range and structural dimensions, with detailed results presented in Table 2. Furthermore, the traditional metal–dielectric–metal high-bandwidth metamaterial absorber proposed in this study exhibits greater bandwidth absorption across a continuous frequency range compared to currently proposed traditional K-band absorbers.

## 6. Conclusions

The proposed metamaterial absorber demonstrates an absorption rate greater than 90% across the frequency range of 20.76–24.20 GHz, with a bandwidth of 3.44 GHz. Three absorption peaks were observed at 21.12 GHz, 22.60 GHz, and 23.86 GHz, with corresponding absorption rates of 96.24%, 98.16%, and 97.10%. The inductance and capacitance were calculated based on transmission line theory, and the simulation was validated through an equivalent circuit model. A detailed analysis of the effective parameters of the metamaterial absorber was conducted, confirming the feasibility of its absorption characteristics. The absorption properties were analyzed and elaborated upon in terms of the induced electric field, magnetic field, and surface current distribution. Additionally, the polarization-sensitive characteristics of the structure were investigated, simulating its absorption rates under oblique incidence at various polarization angles. The study revealed that the absorber maintains good performance across continuous frequencies at different polarization angles, exhibiting strong point absorption under oblique incidence. Finally, practical testing of the metamaterial absorber was performed, which was completely consistent when compared with the simulation results, demonstrating the structure’s high bandwidth absorption characteristics for electromagnetic waves. This design features miniaturization and ultra-wideband absorption capabilities, making it a strong candidate for applications in K-band secure communications, sensing, and filtering. It can be effectively utilized in the field of wireless communication for ultra-wideband electromagnetic wave absorption and single point absorption.

## Figures and Tables

**Table 1:** Design parameters of proposed absorber unit cell.

Parameter	Dimension (mm)
P	9
d	0.5
R2	2.5
b	1.061
L1	7.75
m	0.58
R3	1.4
L2	6.51
R1	2.9
R4	0.9
h	1.575

**Table 2:** Comparison between the proposed metamaterial absorber and related research.

Reference	Year	Physical Dimensions (mm × mm)	Frequency Band	Substrate Material	Resonant Frequency (GHz)
[28]	2023	13.24 × 13.24 × 2	X, Ku and K	FR-4	8.10, 15.39, 19.7
[29]	2020	9 × 9 × 1.6	X, Ku and K	FR-4	11.23, 14.18, 17.37, 19.18
[30]	2022	8 × 8 × 0.65	X, Ku and K	FR-4	8.0, 13.1, 16.08, 19.2
[31]	2021	10 × 10 × 0.16	K	FR-4	21.4–21.8, 23.84–24.24
[35]	2022	10 × 10 × 1.6	Ku and K	FR-4	12.62, 14.12, 17.53, 19.91
[36]	2023	8 × 8 × 0.58	C, X and K	Rogers RO4003C	6.24, 10.608, 18.624
[37]	2024	10 × 10 × 0.1	Ku and K	FR-4	12.06, 19.07
Proposed	2024	9 × 9 × 1.575	K	FR-4	20.76–24.20

## Data Availability

The original contributions presented in the study are included in the article; further inquiries can be directed to the corresponding authors.
